# Health Spending Follows Pace of Population Aging: Challenges Lying Ahead of the Largest Western Balkan Market

**DOI:** 10.3389/fpubh.2015.00058

**Published:** 2015-04-20

**Authors:** Milica Stojkovic, Olivera Milovanovic

**Affiliations:** ^1^Faculty of Medical Sciences, University of Kragujevac, Kragujevac, Serbia; ^2^Department of Pharmacy, Faculty of Medical Sciences, University of Kragujevac, Kragujevac, Serbia

**Keywords:** Serbia, expenditure, health, spending, population, aging, medical care

## Origins of Population Aging in Serbia

Humanity has almost won its battle with many infectious diseases, thus increasing longevity, but is now confronted with challenges arising from population aging (1). A blessing turned into a curse as modern societies began struggling with prosperity diseases proliferation (2). Such obstacles are notable in the largest Western Balkan country (1). One-fifth of the Serbian population is aged 65, where that age group holds 8% globally (3). Serbia has a negative population growth and descending fertility rates, which places its population among the oldest in Europe (4). Following these trends, it is estimated that population older than 75 will make up a majority in the next two decades forming a T shaped age pyramid (5). Within the broader South East European region, population aging trend is clearly present for decades and shows clear signs of acceleration (6).

This phenomenon originated from tumultuous changes in terms of political and economic stability affecting the Balkans in past decades. During the civil war in the 1990s, there has been an exodus of refugees from former parts of Yugoslavia to Serbia. This influx of people accounted for 5% of the total Serbian population count, but left no positive mark on the overall fertility rates due to similar reproductive behavior of internally displaced people (7). Destitution caused by sanctions placed by the Security Council of the UN forced indigenous people to embark on massive emigrations toward richer and more viable economies. Additional impacts on renewal of demographic potential were “brain drain” consisting of almost 50% of skilled emigrants younger than 40 (8).

Difficulties in finding a partner of the opposite sex are also noteworthy. Even though the ratio of men and women is even at reproductive age, the proportion of each gender in rural and urban settings is significantly off-balance (9). Also, the age for women when couples decide on having their first child has shifted from 25 in 2001 to 27 in 2011 (5).

## Local Health Expenditure Trends in Past Two Decades

The Serbian health care system relies financially on Republican Health Insurance Fund (10) that is supported by taxation imposed on the employed population (11). Unfortunately, it is susceptible to budget shortages caused by the growing number of pensioners (12), rise of prosperity diseases, and inefficient allocation of available financial resources for health care (13). Total expenditure on health marked a 45.5% increase for 1995–2010 (Table 1), and the main culprit stigmatized for devouring limited resources were prosperity diseases. Repercussions of an inadequate health care system are weighing on low-income citizens, a sicker population with greater pressure on home budget (14), as total out-of-pocket expenditure for patients rose almost seven times in the last two decades (Table 1).

**Table 1:** **Core population aging indicators for Serbia and expenditure data 1995–2010**.

	**1995**	**2000**	**2005**	**2010**
**Population aging indicators^a^**				
Total fertility rate (children per woman)	1.96	1.74	1.55	1.41
Population growth rate (average annual rate of population change, %)	1.272	−0.198	−0.625	−0.631
Median age of the total population (years)	34.0	35.2	36.1	37.8
Percentage of elderly aged 65+	11.2	12.9	14.0	13.7
Old-age dependency ratio (ratio of population aged 65+ per 100 population 15–64)	16.9	19.3	20.5	19.8
**Health expenditures^b^**				
Total health expenditure (THE) % Gross Domestic Product (GDP)	7	7	9	11
Total expenditure on health (current PPP int. $ per capita)	$260	$313	$771	$1,183
General government expenditure on health (current PPP int. $ per capita)	$184	$219	$509	$732
Private expenditure on health (current PPP int. $ per capita)	$76	$94	$262	$451
Out of pocket expenditure (current PPP int. $ per capita)	$64	$79	$231	$431

*^a^Data source: United Nations Department of Economic and Social Affairs Population Division*.

*^b^Data source: World Health Organization National Health Accounts Global Expenditure database*.

## National Health System Preparedness to Respond to the Challenge

Serbia is not in an enviable economic position with the economy suffering, the public debt doubling, and no sustainable economic solution in the foreseeable future. Republican Health Insurance Fund should depart from previously centrally planned budget and rely on the evidence-based proposals by the academic milieu that reflects real needs at a local level (10). Global economy has exposed unpredictable downfall since 2007 with recession still taking its toll in Eastern Europe.

Serbia must not make impetuous moves in reformation when compiling with EU standards. Local evidence has shown that patient satisfaction with primary care declines in cases when health care reforms are being implemented at an accelerated pace (15). Comparably, the dentistry policy was a hasty reform adopted from patterns found in rich Western societies. Namely, all but emergency dentistry services for adult patients were paid out-of-pocket leading to diminished access of such care (16). However, one of the beneficial and voluntary actions would be implementation of an official HTA agency even though recent fruitless efforts were devoid of legislature maturity (13). Other than lessons gained from the EU, national authorities should learn from the emerging BRIC economies as they recorded some impressive successes in coping with population aging challenges (17). Japan serves as the prime example of the oldest large nation struggling with the burden of medical care for the elderly citizens. By strengthening mandatory taxation of the working age population, they are achieving a short-term solution and not addressing the impeding issue (11). In Serbia, employers and employees alike undergo mandatory taxation for provision of health insurance to the employee’s family members (18). National health system capacities are not prepared for the aforementioned challenges of aging. Rural network of health care facilities suffers from chronic staff shortages. Insurance coverage of the elderly remains mostly insufficient in terms of reimbursed medicines for most chronic disorders. Affordability of home care is more of a privilege than a commonly resorted aspect of medical services. Catastrophic health expenditures due to serious illness of senior family members are frequently sinking families into poverty. These surmounting challenges will need to be addressed within the framework of national strategies tailored to major public health issues. Strengthening of the family medicine capacities and investment into the cost-effective preventive and screening medical technologies are likely an appropriate long-term solution (19). Governmental legislative support to the proliferation of a rather weak private health sector would relieve public health facilities (20). Serbia may effectively fight these challenges by increasing birth rate, thus obtaining a younger taxable working population, and ultimately supporting the health care budget.

## Conflict of Interest Statement

The Review Editor Nela Zivadin Djonovic declares that, despite being affiliated to the same institution as author Milica Stojkovic, the review process was handled objectively and no conflict of interest exists. The authors declare that the research was conducted in the absence of any commercial or financial relationships that could be construed as a potential conflict of interest.
